# P-139. Characteristics of Veteran Travelers at the Clement J. Zablocki Veterans Affairs Medical Center

**DOI:** 10.1093/ofid/ofae631.344

**Published:** 2025-01-29

**Authors:** Bianca Mulaikal, Nathan Gundacker, Katherine Sherman

**Affiliations:** Medical College of Wisconsin, Milwaukee, Wisconsin; The Medical College of Wisconsin, Milwaukee, WI; Clement J Zablocki VAMC, Milwaukee, Wisconsin

## Abstract

**Background:**

Travel location, demographics, and the type of travel are critical determinants of the pre-travel consultation. Veterans represent a unique subpopulation of the US. They earn higher average salaries than their non-veteran counterpart and have access to free healthcare. This puts them in a position to spend more on personal travel, yet there is a paucity of data on veterans' travel preferences and habits. In this study, we examined veteran travel characteristics to provide information on how best provide pre-travel consultation.

**Methods:**

This is a retrospective chart review of veterans who presented to the Clement J Zablocki Veterans Affairs Medical Center (VAMC) infectious disease clinic for a pre-travel consultation between 7/1/2017 and 4/11/2023.

**Results:**

Of the 181 veterans in this study, 150 were male, 29 were female, and two were unknown. The mean traveler was 55.62 years old. Asia (n=87, 48.1%) was the most frequently traveled continent, followed by Africa (n=47, 26%). Veterans were independently traveling (n=121, 67%) for durations greater than one week (n=164, 91%), with 13.3% traveling for at least 2-3 weeks. Within Asia, 23% (n=20) traveled to the Philippines, 26% (n=23) to Vietnam, and 17% (n=15) traveled to Thailand, of these 67% traveled for 4 weeks or more (n=29, 67%).

**Conclusion:**

According to data collected in 2020 by the United Nations World Tourism Organization (UNWTO), the world's top 10 destinations are all within Europe and North America and account for 40% of all global travel. In addition, most group travel is generally one week long. Comparatively, veterans travel longer, more independently, and to more diverse parts of the world. Veteran travel clinics need to be equipped to discuss risks or longer term travel including international health care, safe sex practices and vaccines such as rabies and Japanese encephalitis.

**Disclosures:**

**All Authors**: No reported disclosures

